# PD-Based Optimal ADRC with Improved Linear Extended State Observer

**DOI:** 10.3390/e23070888

**Published:** 2021-07-13

**Authors:** Zhen Zhang, Jian Cheng, Yinan Guo

**Affiliations:** 1 School of Information and Control Engineering, China University of Mining and Technology, Xuzhou 221116, China; TB18060015B4@cumt.edu.cn (Z.Z.); guoyinan@cumt.edu.cn (Y.G.); 2 Research Institute of Mine Big Data, China Coal Research Institute, Beijing 100013, China; 3 School of Mechanical Electronic and Information Engineering, China University of Mining and Technology (Beijing), Beijing 100083, China

**Keywords:** proportional-derivative, active disturbance rejection control, particle swarm optimization, improved extended state observer

## Abstract

Taking dead-zone nonlinearlity and external disturbances into account, an active disturbance rejection optimal controller based on a proportional-derivative (PD) control law is proposed by connecting the proportional-integral-derivative (PID) control, the active disturbance rejection control (ADRC) and particle swarm optimization (PSO), with the purpose of providing an efficient and practical technology, and improving the dynamic and steady-state control performances. Firstly, in order to eliminate the negative effects of the dead-zone, a class of 2-order typical single-input single-out system model is established after compensating the dead-zone. Following that, PD control law is introduced to replace the state error feedback control law in ADRC to simplify the control design. By analyzing the characteristics of the traditional linear extended state observer, an improved linear extended state observer is designed, with the purpose of improving the estimation performance of disturbances. Moreover, employing PSO with a designed objective function to optimize parameters of controller to improve control performance. Finally, ten comparative experiments are carried out to verify the effectiveness and superiority of the proposed controller.

## 1. Introduction

In industrial control systems, the dead-zone non-linearity of the control actuator directly affects the control performance and even leads to instability [1]. In addition, external disturbance is another main reason that reduces the control performance of the system [2]. Considering the above-mentioned negative factors, designing an efficient and practical control method is of great significance for improving system control performance and meeting industrial requirements.

In the past few decades, model-based control methods have been rapidly developed, such as sliding mode control [3] and backstepping control [4]. However, since the above-mentioned control methods are usually more complicated and their control performances depend on the precision of the model of the system, their applications in actual engineering are limited. To the best of our knowledge, the classical PID still plays a dominating role in industrial control systems due to the fact that it does not depend on a precise system model, and has a simple structure [5,6]. Of course, the good control performance of PID depends on the setting of proportional, integral and derivative gains, which is considered a complicated task in actual engineering applications [7,8]. In fact, the integral term of PID has a better effect on suppressing constant disturbance, but when there is no disturbance, it often makes the dynamic performance of the closed-loop system worse. On the other hand, under time-varying disturbances, its anti-disturbance ability is weak [9]. In particular, for systems suffering from external time-varying disturbances, the gains need to be constantly adjusted to ensure good control performance, which can not be achieved in engineering applications. In addition, for a system with dead-zone nonlinearity, the control performance of a system can not be improved by tuning the gains.

Moreover, ADRC as a practical control method, which was originally proposed by Han in 1998 [10], has received more and more attentions [11,12,13,14,15]. The stability analysis of ADRC is an open challenge for ADRC-based control systems. Aguilar-Iba$\tilde{n}$ez et al. [16] discussed the stability of ADRC for uncertain system via direct Lyapunov method. Following that, the effectiveness of ADRC was verified by successful applications on a ball and rigid triangle system [17] and uncertain second-order flat systems [18]. Without loss of generality, ADRC consists of transition process (TP), extended state observer (ESO), and state error feedback control law (SEFCL). Among them, ESO estimates the disturbance based on the input and output of a controlled process, with the purpose of effectively improving the anti-disturbance ability of a control system. However, there are several parameters in the ESO that need to be determined, and there is a lack of parameter tuning methods for reference in the existing researches. Moreover, its superior estimation accuracy depends on large gains, which amplifies noise, thereby deteriorating control performance, even causing instability [19]. TP produces a desired trajectory with shorter settling time and smaller error in terms of expected signal. According to the difference between output of ESO and that of TP, a SEFCL is designed. Discrete TP is widely employed to avoid high-frequency chattering caused by discretizing continuous one. However, a third- or higher-order discrete TP is hardly to be designed, limiting the application of ADRC [19].

Compared with PID and ADRC, the structure of PID is simpler, which is more conducive to engineering applications. ADRC has stronger anti-disturbance ability and effectively improves system control performance. It is worth noting that ADRC is essentially an improved nonlinear PID control method. SEFCL in ADRC is regarded as a PD control law. On the other hand, the integral term of PID and ESO both play the role of suppressing disturbances [19,20]. Based on this, the organic integration of PID and ADRC is bound to obtain a more efficient controller. Zhong et al. [21] proposed a parameter formula by combining PID and ADRC, with the purpose of improving robustness and tracking performance of a 2-order system. Since the proposed control law rely on the outputs of TP, the method proposed in [21] is not suitable for higher-order systems. Wang et al. [22] proposed a double closed-loop control method based on PID and ADRC to solve the position and attitude control of a quadrotor helicopter system with model uncertainties and disturbances, however, the above-mentioned control method has a complicated structure and many parameters. Liu et al. [23] proposed an ADRC-based fractional-order PID for an active power filter, with the purpose of improving robustness and control performance. However, the design of the control law is complicated and there are many control parameters to be tuned. Ren et al. [24] proposed a back-propagation PID with based on a nonlinear ESO to achieve precise control of wind turbines. The method proposed in [24] used a neural network to optimize only the parameters of the PID, but did not optimize the parameters of the active disturbance rejection controller, which is detrimental to improving the control performance of the system. In addition, the larger gains of ADRC will amplify noise, which may reduce control performance.

The optimal parameter setting of controller has a direct effect on improving the control performance of the system [25]. As a mature algorithm, PSO was originally proposed by Kennedy and Eberhart in 1995 [26]. It is an intelligent optimization algorithm based on the foraging behavior of birds or fish. Due to the advantages of simple implementation and high search efficiency [27,28], it has been applied to the parameter optimization of the control systems [29,30,31,32]. Therefore, PSO is introduced to seek the best parameters of the controller in this paper, which is of great significance to improve the control performance.

In view of this, a class of 2-order typical single-input single-out system model is established after compensating the dead-zone. Following that, PD and PSO are introduced into ADRC, forming a PD-based ADRC optimal controller. This paper has the following fourfold contributions:

1. Establishing a dead-zone compensated model. By introducing a compensation method [1], the influence of the dead-zone nonlinearity on the control system is eliminated.
2. Introducing a PD as the control law. Compared with SEFCL, PD has the advantages of simple design, fewer parameters, and easy application.
3. Designing an improved linear ESO with smaller gains. The proposed observer is established based on the estimated errors of all state variables, with the purpose of enhancing estimation performance for disturbances with smaller gains.
4. Optimizing parameters by PSO with a designed objection function. The controller with the optimal parameters provides better dynamic and steady-state control performances.

The rest of this paper is structured as follows: Section 2 establishes the dead-zone compensated model; Section 3 propose the PD-based ADRC optimal controller; Section 4 provides the comparative experiments, and analysis of the proposed controller. Finally, the whole paper is conclude and future direction is provided in Section 5.

## 2. The Model of a Controlled System

Without loss of generality, a typical 2-order single-input single-output system with dead-zone nonlinearlity and external disturbances is modeled as follows:

(1) $$\left\{\begin{array}{c} {\dot{x}}_{1}=x_{2}\\ {\dot{x}}_{2}=f_{0}\left(x_{1},x_{2},\omega \left(t\right)\right)+b_{0}u_{d}\\ y=x_{1}\\ u_{d}=\mathrm{dz}\left(v\right) \end{array}\right.$$

where $X=\left(x_{1},x_{2}\right)\in R{}^{2}$ is the state vector and can be measured; $u_{d}$ and *y* are the input and output of the controlled system, respectively; *v* represents the original control signal; dz(v) represents the dead-zone nonlinearity of *v*; $f_{0}\left(x_{1},x_{2},\omega \left(t\right)\right)\in R$ represents the unknown factors that depend on *X* and external time-varying disturbance ω(t); $b_{0}$ is a gain.

**Figure 1:**
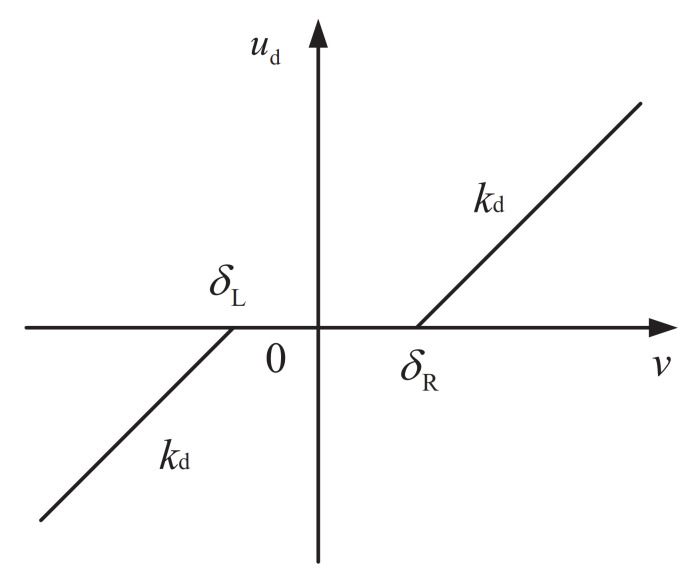
Dead-zone nonlinearity.

The control actuator of an actual system usually has asymmetrical dead-zone nonlinearity [33,34], as shown in Figure 1. $k_{d}$ is the gain, $\delta_{L}$ and $\delta_{R}$ are the uncertain boundary parameters of the dead-zone.

**Definition** **1.** *Let $\delta ={\left[\delta_{R},\delta_{L}\right]}^{T}$, $\hat{\delta }={\left[{\hat{\delta }}_{R},{\hat{\delta }}_{L}\right]}^{T}$ and $\tilde{\delta }=\hat{\delta }-\delta$ are the corresponding estimation and estimated error of δ, respectively.*

After using the dead-zone compensator proposed by Lewis [1], $u_{d}$ in Equation (1) can be represented as follows:

(2) $$u_{d}=\mathrm{dz}\left(v\right)=k_{d}\left[u+{\tilde{\delta }}^{T}\left(\bar{\alpha }-\bar{\beta }\right)\right]$$

In the above formula, *u* is the control variable to be designed, $\bar{\alpha }={\left[\alpha ,\;1-\alpha \right]}^{T}$, $\bar{\beta }=[\alpha \xi \left({\tilde{\delta }}_{R}\right),(1-\alpha )\xi \left({\tilde{\delta }}_{L}\right){]}^{T}\mathrm{sat}\left(u\right)$, where α=1 as u≥0 and α=0 as u<0. ξ(·) represents a unit step function, sat(*u*) is represented as follows:

(3) $$\begin{array}{c} \mathrm{sat}(u)=\left\{\begin{array}{cc} 0 & u>-{\tilde{\delta }}_{R}\\ 1+u/{\tilde{\delta }}_{R} & 0<u\leq -{\tilde{\delta }}_{R}\\ 1+u/{\tilde{\delta }}_{L} & -{\tilde{\delta }}_{L}<u\leq 0\\ 0 & u\leq -{\tilde{\delta }}_{L} \end{array}\right. \end{array}$$

Let $b=b_{0}k_{d}$, then, substituting Equation (2) into Equation (1), one has

(4) $$\left\{\begin{array}{c} {\dot{x}}_{1}=x_{2}\\ {\dot{x}}_{2}=f_{0}\left(x_{1},x_{2},\omega \left(t\right)\right)+b{\tilde{\delta }}^{T}\left(\bar{\alpha }-\bar{\beta }\right)+bu\\ y=x_{1} \end{array}\right.$$

Let $f_{0}\left(x_{1},x_{2},\omega \left(t\right)\right)+b{\tilde{\delta }}^{T}\left(\bar{\alpha }-\bar{\beta }\right)$ represent the “total disturbance”, denoted as $f(x_{1},x_{2},\omega \left(t\right),\delta_{L},\delta_{R})$, then, Equation (4) can be transformed as follows:

(5) $$\left\{\begin{array}{c} {\dot{x}}_{1}=x_{2}\\ {\dot{x}}_{2}=f\left(x_{1},x_{2},\omega \left(t\right),\delta_{L},\delta_{R}\right)+bu\\ y=x_{1} \end{array}\right.$$

## 3. The Proposed PD-Based ADRC Optimal Control Method

**Figure 2:**
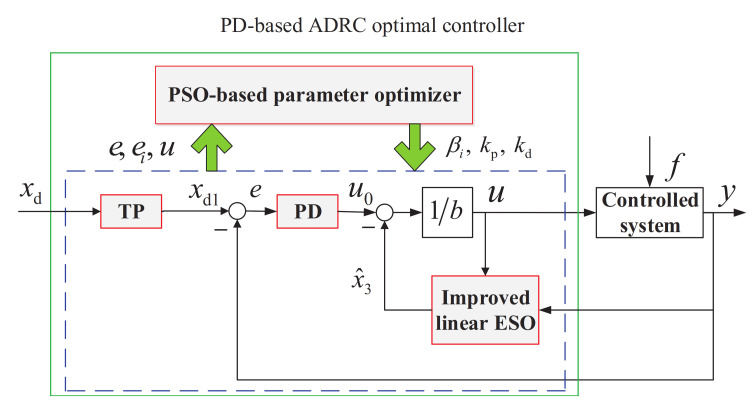
The framework of the proposed controller.

In this paper, taking dead-zone nonlinearity, and external disturbance into account, a PD-based ADRC optimal controller is proposed by combining PD, ADRC and PSO, with the purpose of simplifying the design as much as possible while improving the control performance. As shown in Figure 2, the framework of the proposed control method includes four parts: (1) TP: producing an expected tracking trajectory; (2) PD: providing a simpler and easier-to-apply control law; (3) Improved linear ESO: estimating the total disturbance more efficiently with smaller gains; (4) PSO-based parameter optimizer: producing the optimal parameters for controller.

### 3.1. Transition Process

When the system responds quickly, the larger initial error and step error caused by the step reference signal may cause overshoot. To solve the above problem, a TP is introduced to generate a smooth and continuous ideal tracking trajectory. In addition, since the system chattering that may be caused by continuous TP, a discrete 2-order TP is used as follows [19]:

(6) $$\left\{\begin{array}{c} {x_{d}}_{1}\left(k+1\right)={x_{d}}_{1}\left(k\right)+h{x_{d}}_{2}\left(k\right)\\ {x_{d}}_{2}\left(k+1\right)={x_{d}}_{2}\left(k\right)+hu_{1} \end{array}\right.$$

where $x_{d1}$ and $x_{d2}$ are the outputs of the TP; $k\in N^{+}$, *h* is the integration step, $u_{1}$ represents a fast control function proposed by Han [19], which is described as follows:

(7) $$\left\{\begin{array}{c} d=rh_{0},\;d_{0}=h_{0}d,\;h_{0}>h\\ y^{\prime }={x_{d}}_{1}-x_{d}+h_{0}{x_{d}}_{2},\;a_{0}=\sqrt{d^{2}+8r\left|y^{\prime }\right|}\\ \begin{array}{c} a_{1}=\left\{\begin{array}{cc} {x_{d}}_{2}+\frac{a_{0}-d}{2} & |y^{\prime }|>d_{0}\\ {x_{d}}_{2}+\frac{y^{\prime }}{h_{0}} & |y^{\prime }|\leq d_{0} \end{array}\right. \end{array}\\ \begin{array}{c} u_{1}=-\left\{\begin{array}{cc} r\mathrm{sgn}(a_{1}) & |a_{1}|>d\\ r\frac{a_{1}}{d} & |a_{1}|\leq d \end{array}\right. \end{array} \end{array}\right.$$

In the above formula, *r* is the speed factor and adjusts the tracking speed of $x_{d}$ to $x_{d1}$. $h_{0}$ is a new variable independent of the integration step length *h*. Generally, $h_{0}>h$, with the purpose of eliminating the overshoot, and avoiding amplifying noise in the differential signal. $d_{0}$ depends on *r* and $h_{0}$. $a_{1}$ is related to $x_{d2}$, $a_{0}$, *d*, $y^{\prime }$ and $h_{0}$. In order to provide a fast and accurate response for control system, the key parameters of the proposed transition process, such as *r* and $h_{0}$, are tuned online [19]. Based on them, we can determine the values of *d*, $d_{0}$, $y^{\prime }$, $a_{0}$ and $a_{1}$.

### 3.2. PD Control Law

In Section 3.1, a 2-order discrete TP is employed, represented by Equation (6), with the purpose of simplifying design and facilitating engineering applications. Denote $x_{d}$ as a set reference signal, and $x_{d1}$ as a desired reference signal of a controlled variable produced by TP. Based on this, $e=x_{d1}-y$ is defined as a tracking error, where $x_{d1}$ is the output of transition process, and $y=x_{1}$ is the output of the system. Then, we obtain the derivative of *e* as $\dot{e}={\dot{x}}_{d1}-x_{2}$. PD is employed as the control law to provide a control signal, represented by $u_{0}$, for the controlled system expressed by Equation (5) as follows:

(8) $$u_{0}=k_{p}e+k_{d}\dot{e}$$

where $k_{p}$, $k_{d}$ are the gains of PD-based control law.

### 3.3. An Improved Linear ESO

ESO can estimate the disturbance in real time based on the input and output of the system without any information about the disturbance. Following that, the estimated disturbance is compensated by combining a control law to achieve the purpose of improving the control performance. The linear ESO has the advantages of simple design, fewer parameters, and suitability in engineering applications [19].

Denote $x_{3}$ as the extended state variable of *f* in Equation (5). For the system, represented by Equation (5), the traditional linear ESO is described as follows:

(9) $$\left\{\begin{array}{c} e_{1}={\hat{x}}_{1}-x_{1}\\ {\dot{\hat{x}}}_{1}={\hat{x}}_{2}-\beta_{1}e_{1}\\ {\dot{\hat{x}}}_{2}={\hat{x}}_{3}-\beta_{2}e_{1}+bu\\ {\dot{\hat{x}}}_{3}=-\beta_{3}e_{1} \end{array}\right.$$

where $\beta_{i}>0$, i=1,2,3 are the gains of the linear ESO. ${\hat{x}}_{i}$ is an estimation of $x_{i}$, $e_{1}$ is the estimated error of $x_{1}$.

The traditional linear ESO adjusts the estimation increment, represented by ${\dot{\hat{x}}}_{i}$, in terms $e_{1}$. However, once ${\hat{x}}_{1}$ is close to $x_{1}$ enough, $e_{1}$ is too small to achieve the approximation of ${\hat{e}}_{\tau }$ to $e_{\tau }$, τ=2,3. To tackle the problem, the parameters in the estimated model, such as $\beta_{i}$, are set to the larger values. However, large $\beta_{i}$ may amplify noise, causing chattering, even instability [19]. In view of this, we propose an improved linear ESO with smaller gains to obtain better estimation performance, in which estimation errors of all state variables, represented by $e_{j}={\hat{x}}_{j}-x_{j}$, j=1,2, are introduced to Equation (9), instead of $e_{1}$:

(10) $$\left\{\begin{array}{c} {\dot{\hat{x}}}_{1}={\hat{x}}_{2}-\beta_{1}e_{1}\\ {\dot{\hat{x}}}_{2}={\hat{x}}_{3}-\beta_{2}e_{2}+bu\\ {\dot{\hat{x}}}_{3}=-\beta_{3}e_{2} \end{array}\right.$$

In the above formula, $\beta_{1}>0$, $\beta_{2}>0$ and $\beta_{3}>0$ are adjusted online [19], with the purpose of ensuring higher estimation accuracy of the observer. $e={\left[e_{1},e_{2},e_{3}\right]}^{T}$ is an estimated error vector, where $e_{3}={\hat{x}}_{3}-x_{3}$. Define ${\dot{x}}_{3}=\dot{f}=f_{1}$, the dynamic estimated error is achieved after integrating Equation (5) with Equation (10).

(11) $$\dot{e}=Ae+Bf_{1}$$

where, $A=\left[\begin{array}{ccc} -\beta_{1} & 1 & 0\\ 0 & -\beta_{2} & 1\\ 0 & -\beta_{3} & 0 \end{array}\right]$, $B=\left[\begin{array}{c} 0\\ 0\\ -1 \end{array}\right]$.

In actual engineering, the “total disturbance” expressed by *f* is usually bounded, $x_{3}=f$, consequently, is bounded. Since $\beta_{i}>0$, *A* is a Hurwitz matrix. According to Hurwitz stability theory [35], the differential equation expressed by Equation (11) is stable. Therefore, the improved linear ESO is stable and its estimated errors are bounded.

Moreover, the proposed improved linear ESO can be extended to a (n+1)-order observer, expressed by Equation (13), to estimate the “total disturbance” of a class of *n*-order single-input single-output systems, represented by Equation (12), and its stability can also be guaranteed:

(12) $$\left\{\begin{array}{c} {\dot{x}}_{1}=x_{2}\\ {\dot{x}}_{2}=x_{3}\\ \;\;\;\;\;\vdots \\ {\dot{x}}_{n}=f+bu\\ y=x_{1} \end{array}\right.$$

The improved (n+1)-order linear ESO for the above *n*-order systems is designed as follows:

(13) $$\left\{\begin{array}{c} {\dot{\hat{x}}}_{1}={\hat{x}}_{2}-\beta_{1}e_{1}\\ {\dot{\hat{x}}}_{2}={\hat{x}}_{3}-\beta_{2}e_{2}\\ \;\;\;\;\;\vdots \\ {\dot{\hat{x}}}_{n}={\hat{x}}_{n+1}-\beta_{n}e_{n}+bu\\ {\dot{\hat{x}}}_{n+1}=-\beta_{n+1}e_{n} \end{array}\right.$$

where ${\hat{x}}_{\mu }$ is an estimation of $x_{\mu }$, μ=1,⋯,n+1; $\beta_{\eta }$, η=1,⋯,n is the gain of the improved linear ESO; $e_{\eta }={\hat{x}}_{\eta }-x_{\eta }$ is the estimated error of state variable. In order to ensure the higher estimation accuracy of the observer, $\beta_{1}>0$, ⋯, $\beta_{n+1}$ can be selected through online adjustment [19].

### 3.4. Design of the PD-Based ADRC Optimal Controller

Through the organic combination of PD, improved linear ESO and PSO, a PD-based ADRC optimal controller is developed. In the proposed controller, PD, represented by $u_{0}=k_{p}e+k_{d}\dot{e}$, is integrated with ${\hat{x}}_{3}$ obtained from the improved linear ESO, the final output of the proposed controller is thus obtained as follows:

(14) $$u=\frac{u_{0}-{\hat{x}}_{3}}{b}$$

**Remark** **1.** *The proposed controller, represented by Equation (14), can be transformed into $u=k_{p0}e+k_{i0}{\hat{x}}_{3}+k_{d0}\dot{e}$, where $k_{p0}=\frac{k_{p}}{b}$, $k_{i0}=-\frac{1}{b}$, and $k_{d0}=\frac{k_{d}}{b}$. The integral term of PID and $k_{i0}{\hat{x}}_{3}$ of the proposed controller are both to suppress the influence of the disturbances on the system. Therefore, the proposed controller can be regarded as a kind of improved PID controller with stronger robustness, which is beneficial to its application in engineering. According to Remark 1, it can be obtained that the proposed control method is also suitable for high-order systems.*

In the above controller, $\beta_{i}$, $k_{p}$, and $k_{d}$ need to be tuned to ensure good control performance. This section employs PSO and a designed objective function jointly to construct a PSO-based parameter optimizer. In the optimizer, an individual is encoded as $X_{j}=\left(\beta_{i},k_{p},k_{d}\right)$. Assuming that a particle swarm contains *m* particles, and the dimension of each particle is *D*. The position and velocity of *k*-th particle are denoted as $X_{k}=\left(X_{k1},\cdots ,X_{kD}\right),k=1,\cdots ,m$ and $v_{k}=\left(v_{k1},\cdots ,v_{kD}\right)$. The optimal positions reached by the *k*-th particle and the entire particle swarm are expressed by $p_{k}=\left(p_{k1},\cdots ,p_{kD}\right)$ and $p_{g}=\left(p_{g1},\cdots ,p_{gD}\right),g=1,\cdots ,m$. During the N+1 iteration, each particle updates its velocity and position in the following manner:

(15) $$v_{kd}^{N+1}=\chi v_{kd}^{N}+c_{1}r_{1}\left(p_{kd}^{N}-x_{kd}^{N}\right)+c_{2}r_{2}\left(p_{gd}^{N}-x_{gd}^{N}\right)$$

(16) $$x_{kd}^{N+1}=x_{kd}^{N}+v_{kd}^{N+1}$$

In the above formulas, $N\in N^{+}$; d=1,2,⋯,D; χ represents the inertia coefficient; $c_{1}$ and $c_{2}$ are two acceleration factors; $r_{1}\in \left[0,1\right]$ and $r_{2}\in \left[0,1\right]$ are two random numbers. Considering the control performance in terms of $e_{i}$, *e*, and *u*, the objective function is designed as follows:

(17) $$J=\int_{0}^{t}\left(\sum_{i=1}^{n+1}\left|e_{i}\right|+\left|e\right|+\left|u\right|\right)dt$$

The PSO-based parameter optimization process may be broken down in the following steps.

Step 1: Initializing the initial position and velocity of all particles;

Step 2: Calculating the fitness value of each particle;

Step 3: Updating the local and global optima by Equation (17);

Step 4: Updating the position and velocity of each particle according to Equations (15) and (16);

Step 5: Judging whether the iteration reaches its maximum, if yes, stop searching and output the global optimum; otherwise, jump to step 2.

## 4. Experimental Results and Analysis

In order to verifiy the effectiveness and superiority of the proposed PD-based ADRC optimal controller, ten comparative experiments are carried out by MATLAB 2016b on an Intel (R) Core (TM) i5-6500 CPU @ 3.20 GHZ 3.19 GHZ 4.00 GB RAM, Windows 10 platform. Let us consider the following controlled system:

(18) $$\left\{\begin{array}{c} {\dot{x}}_{1}=x_{2}\\ {\dot{x}}_{2}=f\left(x,\omega \left(t\right)\right)+bu\\ y=x_{1} \end{array}\right.$$

where f=5 as t∈[0,5]s, f=−1 as t∈(5,10]s, f=6 as t∈(10,15]s, f=18 as t∈(15,20]s; b=133; $x_{d}$ = 0.5 as t∈ [0,10)s; $x_{d}$ = 1 as t∈ [10,20]s.

In the proposed controller, h=0.002, $h_{0}=0.01$, r=1, ω = 1, $c_{1}$ = 2, $c_{2}$ = 2, and the detailed values of optimized parameters are obtained as follows: $k_{p}=9.56$, $k_{d}=112.66$, $\beta_{1}=3.52$, $\beta_{2}=55.26$, $\beta_{3}=1060.89$. Moreover, nine control methods are used as comparison methods to verify the superiority of the proposed controller, as follows.

(1) Traditional PD (TPD). Let $k_{p}$, $k_{d}$ are proportional and derivative gains of PD, respectively.

(19) $$u=k_{p}e+k_{d}\dot{e}$$

where $k_{p}=0.6$, $k_{d}=0.02$.

(2) Traditional PID (TPID). Let $k_{p}$, $k_{i}$, $k_{d}$ are proportional, integral and derivative gains of TPID, respectively.

(20) $$u=k_{p}e+k_{i}\int_{0}^{t}edt+k_{d}\dot{e}$$

where $k_{p}=0.6$, $k_{i}=0.4$, $k_{d}=0.02$.

(3) Traditional PID with a 2-order discrete TP (TPID-TP). Let $k_{p}$, $k_{i}$, $k_{d}$ are proportional integral and derivative gains of TPID-TP, respectively.

(21) $$u=k_{p}e+k_{i}\int_{0}^{t}edt+k_{d}\dot{e}$$

where h=0.002, $h_{0}=0.01$, r=1, $k_{p}=0.6$, $k_{i}=0.4$, $k_{d}=0.02$.

(4) Linear ADRC with linear ESO (LADRC-LESO).

(22) $$u=\frac{u_{0}-{\hat{x}}_{3}}{b}$$

where $u_{0}=10\left(x_{d1}-{\hat{x}}_{1}\right)+100\left(x_{d2}-{\hat{x}}_{2}\right)$; $\beta_{1}=100$, $\beta_{2}=3000$, $\beta_{3}$ = 10,000.

(5) Nonlinear ADRC with linear ESO (NADRC-LESO).

(23) $$u=\frac{u_{0}-{\hat{x}}_{3}}{b}$$

where $u_{0}=10fal\left(\left(x_{d1}-{\hat{x}}_{1}\right),\alpha_{1},\delta \right)+100fal\left(\left(x_{d2}-{\hat{x}}_{2}\right),\alpha_{1},\delta \right)$; $\beta_{1}=100$, $\beta_{2}=3000$, $\beta_{3}$ = 10,000, $\alpha_{1}=0.5$, $\alpha_{2}=0.75$, δ=0.01, and

(24) $$\begin{array}{c} \mathrm{fal}(*,\alpha_{j},\delta )=\left\{\begin{array}{cc} {\left|*\right|}^{\alpha_{j}}\mathrm{sgn}\left(*\right) & \left|*\right|>\delta \\ \frac{*}{\delta^{\left(1-\alpha_{j}\right)}} & \left|*\right|\leq \delta \end{array}\right. \end{array}$$

(6) Linear ADRC with improve linear ESO (LADRC-ILESO).

(25) $$u=\frac{u_{0}-{\hat{x}}_{3}}{b}$$

where $u_{0}=10\left(x_{d1}-{\hat{x}}_{1}\right)+100\left(x_{d2}-{\hat{x}}_{2}\right)$; $\beta_{1}=3.52$, $\beta_{2}=55.26$, $\beta_{3}=1060.89$.

(7) Nonlinear ADRC with improve linear ESO (NADRC-ILESO).

(26) $$u=\frac{u_{0}-{\hat{x}}_{3}}{b}$$

where $u_{0}=10fal\left(\left(x_{d1}-{\hat{x}}_{1}\right),\alpha_{1},\delta \right)+100fal\left(\left(x_{d2}-{\hat{x}}_{2}\right),\alpha_{1},\delta \right)$; $\beta_{1}=3.52$, $\beta_{2}=55.26$, $\beta_{3}=1060.89$, $\alpha_{1}=0.5$, $\alpha_{2}=0.75$, δ=0.01.

(8) PD with linear ESO and a 2-order discrete TP (PD-LESO-TP).

(27) $$u=\frac{u_{0}-{\hat{x}}_{3}}{b}$$

where h=0.002, $h_{0}=0.01$, r=1, $u_{0}=k_{p}e+k_{d}\dot{e}$; $k_{p}=0.6$, $k_{d}=0.02$, $\beta_{1}=100$, $\beta_{2}=3000$, $\beta_{3}$ = 10,000.

(9) PD with improve linear ESO and a 2-order discrete TP (PD-ILESO-TP).

(28) $$u=\frac{u_{0}-{\hat{x}}_{3}}{b}$$

where h=0.002, $h_{0}=0.01$, r=1, $u_{0}=k_{p}e+k_{d}\dot{e}$; $k_{p}=0.6$, $k_{d}=0.02$, $\beta_{1}=3.52$, $\beta_{2}=55.26$, $\beta_{3}=1060.89$.

**Figure 3:**
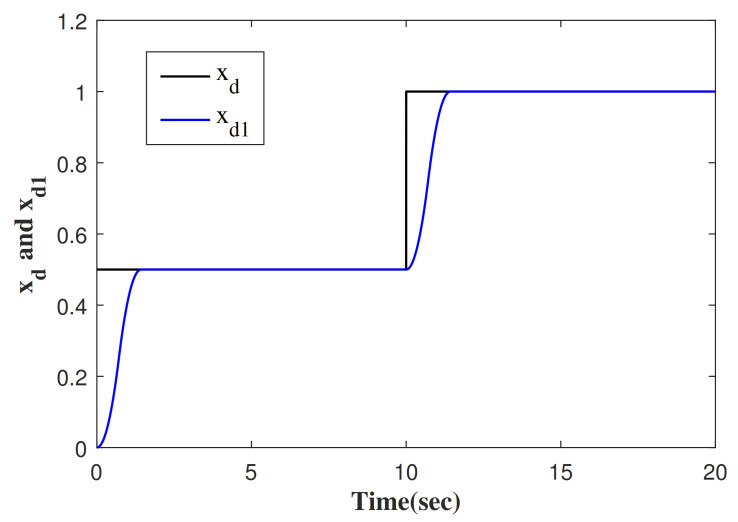
The response of $x_{d1}$.

**Figure 4:**
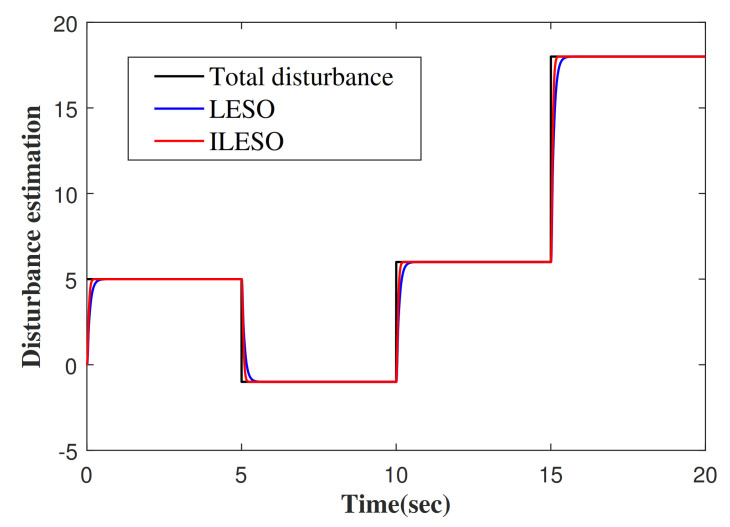
The estimation of disturbance.

The desired tracking trajectory generated by TP is shown in Figure 3. In the actual control system, there may be step disturbances that affect the performance of the system. In view of this, the total disturbance, as shown in Figure 4, is chosen to verify the effectiveness of the proposed control method. The approximate responses of the traditional linear ESO (LESO) and the improved linear ESO (ILESO) to the “total disturbance” are shown in Figure 4.

**Figure 5:**
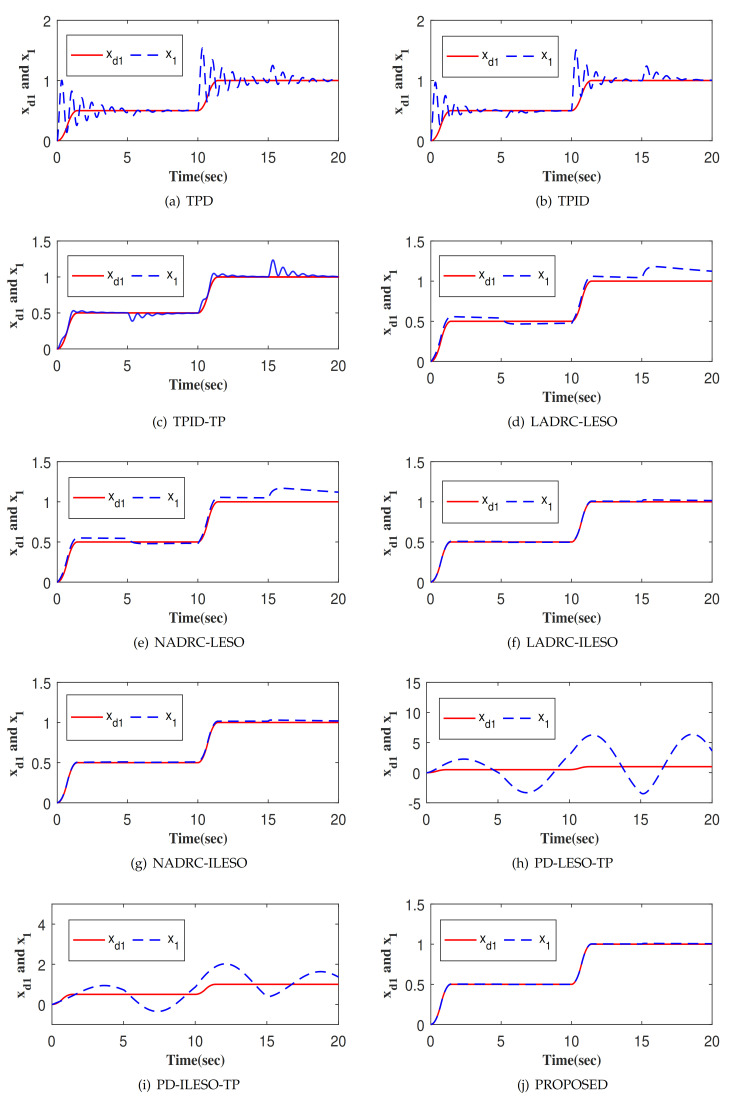
The tracking response of different control methods.

**Figure 6:**
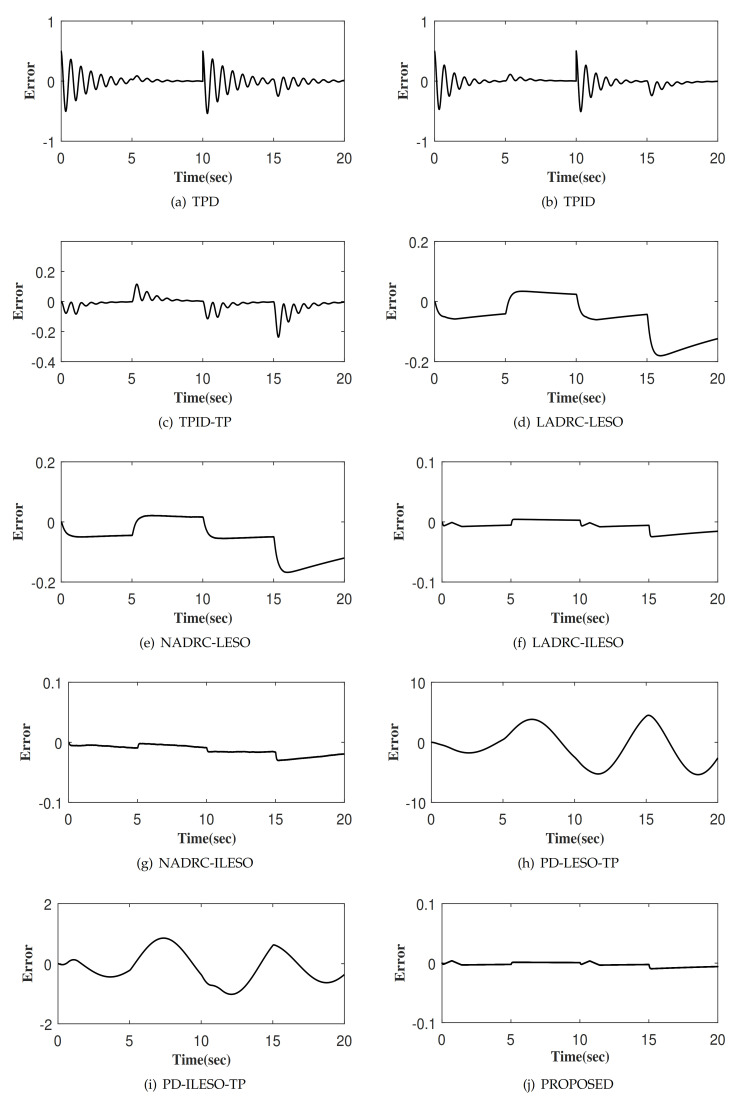
The tracking error of different control methods.

**Figure 7:**
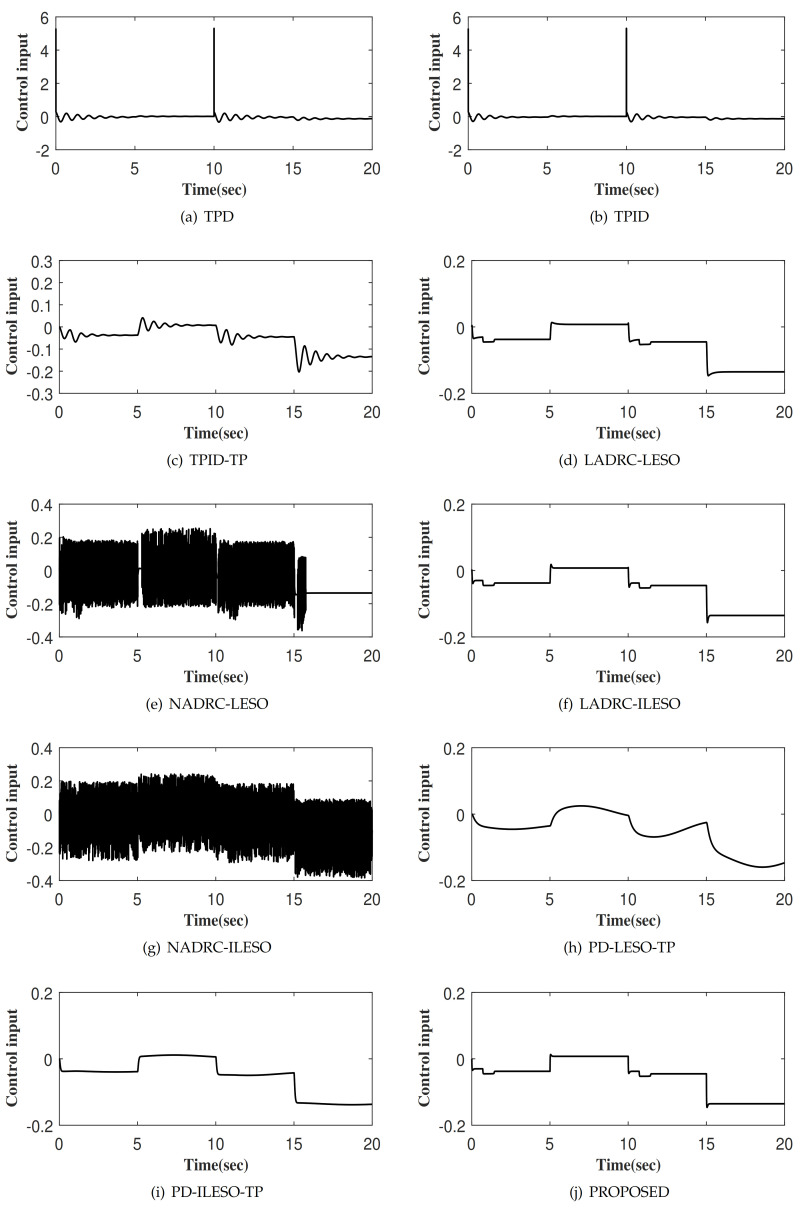
The control input of different control methods.

The tracking responses of ten control methods to the output of the TP, denoted as $x_{d1}$ is shown in Figure 5. Among them, Figure 5j shows the step response of $x_{1}$ for the proposed control. Obviously, the proposed control method achieves fast tracking $x_{d1}$ without overshoot. Figure 6 and Figure 7 depict the tracking errors and control inputs of ten controllers. Moreover, four performance indexes of tracking error, including maximum absolute error (MAAE), mean absolute error (MEAE), standard deviation of absolute error (SDAE), the integral time absolute error (ITAE), as listed in Table 1. Four performance indexes of control input, including maximum absolute control input (MAACI), mean absolute control input (MEACI), standard deviation of absolute control input (SDACI), and the integral time absolute control input (ITACI) are also employed to fully analyze the control performances, as listed in Table 2. Comparative experiment results show that the proposed control method achieves the smallest tracking error with the smallest control input, which verifies its effectiveness and superiority. Specifically, by comparing with nine control methods, the following conclusions are obtained: (1) The control method can improve the control performance with the assistance of TP strategy, which verifies the effectiveness and rationality of TP. (2) The control method can significantly improve the control performance with the aid of the ILESO strategy with small gains, which verifies the effectiveness and rationality of ILESO. (3) The control method with optimal control parameters has better control performance, which verifies the effectiveness and rationality of the parameter optimization strategy. Furthermore, the performance of the controllers PD-ILESO-TP and PD-LESO-TP with parameters set according to the traditional control method is extremely poor, and the proposed parameter optimization strategy can find the optimal parameters for the controller to achieve better control performance. For non-linear control methods NADRC-LESO and NADRC-ILESO, because their control laws show the non-smooth characteristics, high-frequency chattering occurs in the control input, which is not conducive to engineering applications. The proposed method is a litter more complicated than TPD, TPID and TPID-TP, but the control performance is superior to them. The proposed control method is not only simpler than LADRC-LESO, NADRC-LESO, LADRC-ILESO, and NADRC-ILESO, but also has superior control performance. The control method proposed in this paper not only obtains the best control performance, but also is suitable for high-oeder systems, and is a practical control method with strong competitiveness.

## 5. Conclusions

For a typical 2-order single-input single-output system subjected to dead-zone nonlinearlity and external disturbances, a PD-based ADRC optimal controller is proposed by connecting the PD, ILESO and PSO-based parameter optimizer. Different from the traditional PID, the ILESO of the proposed control method greatly improves the anti-disturbance ability of the system with smaller gains. Unlike ADRC, the proposed control method is simpler and more effective, and is suitable for high-order systems. In addition, the proposed parameter optimization strategy can seek the optimal control parameters, which not only provides a parameter setting method, but also further improves the system control performance. The comparative experiment results verified the effectiveness and superiority of the proposed control method. In conclusion, the proposed control method provides an efficient control technology for industrial engineering. The ILESO in this paper is proposed based on the fact that the system state variables can be completely measured, which has certain limitations. Therefore, considering that the state variables of the system are not completely measurable, designing an efficient observer for PD-based control method is our future work.

## Figures and Tables

**Table 1:** Comparison of the tracking error among different control methods.

Control Methods	MAAE	MEAE	SDAE	ITAE
TPD	0.5379	0.0740	0.1004	1.4800
TPID	0.5072	0.0568	0.0879	1.1354
TPID-TP	0.2369	0.0271	0.0361	0.5427
LADRC-LESO	0.1804	0.0692	0.0499	1.3842
NADRC-LESO	0.1679	0.0641	0.0489	1.2816
LADRC-ILESO	0.0246	0.0088	0.0067	0.1768
NADRC-ILESO	0.0303	0.0131	0.0083	0.2615
PD-LESO-TP	5.3777	2.5274	1.6213	50.5480
PD-ILESO-TP	1.0193	0.4437	0.2779	8.8744
PROPOSED	0.0094	0.0034	0.0026	0.0672

**Table 2:** Comparison of the control input among different control methods.

Control Methods	MAACI	MEACI	SDACI	ITACI
TPD	5.3067	0.0776	0.1012	1.5522
TPID	5.3075	0.0706	0.0969	1.4118
TPID-TP	0.2031	0.0571	0.0490	1.1415
LADRC-LESO	0.1469	0.0566	0.0478	1.1309
NADRC-LESO	0.3619	0.0947	0.0619	1.8946
LADRC-ILESO	0.1568	0.0565	0.0478	1.1290
NADRC-ILESO	0.3836	0.0985	0.0733	1.9699
PD-LESO-TP	0.1596	0.0610	0.0492	1.2193
PD-ILESO-TP	0.1384	0.0574	0.0472	1.1474
PROPOSED	0.1462	0.0564	0.0478	1.1282
